# Insight into the dynamics of second grade hybrid radiative nanofluid flow within the boundary layer subject to Lorentz force

**DOI:** 10.1038/s41598-021-84144-6

**Published:** 2021-03-01

**Authors:** Muhammad Jawad, Anwar Saeed, Asifa Tassaddiq, Arshad Khan, Taza Gul, Poom Kumam, Zahir Shah

**Affiliations:** 1 Department of Mathematics, Abdul Wali Khan University, Mardan, 23200 Khyber, Pakhtunkhwa Pakistan; 2 Department of Basic Sciences and Humanities, College of Computer and Information Sciences, Majmaah University, Al-Majmaah, 11952 Saudi Arabia; 3 College of Aeronautical Engineering, National University of Sciences and Technology (NUST), Sector H-12, Islamabad, 44000 Pakistan; 4 Department of Mathematics, City University of Science and Information Technology, Peshawar, 25000 Khyber, Pakhtunkhwa Pakistan; 5 Fixed Point Research Laboratory, Fixed Point Theory and Applications Research Group, Center of Excellence in Theoretical and Computational Science (TaCS-CoE), Faculty of Science, King Mongkut’s University of Technology Thonburi (KMUTT), 126 Pracha Uthit Rd., Bang Mod, Thung Khru, Bangkok, 10140 Thailand; 6 Center of Excellence in Theoretical and Computational Science (TaCS-CoE), Faculty of Science, King Mongkut’s University of Technology Thonburi (KMUTT), 126 Pracha Uthit Rd., Bang Mod, Thung Khru, Bangkok, 10140 Thailand; 7 Department of Mathematical Sciences, University of Lakki Marwat, Lakki Marwat, 28420 Khyber Pakhtunkhwa Pakistan

**Keywords:** Mathematics and computing, Applied mathematics

## Abstract

The magnetohydrodynamic hybrid second-grade nanofluid flow towards a stretching/shrinking sheet with thermal radiation is inspected in current work. Main concern of current investigation is to consider hybrid $Al_{2} O_{3} - Cu$ nanofluid which is perceived by hanging two dissimilar kinds of nanoparticles known as alumina and copper within the base fluid. The fluid motion is produced by non-linear stretching/shrinking sheet. The modeled equations which comprise of energy, motion and continuity equations are changed into dimensionless form using group of similar variables. To determine the solution of transformed problem, the Homotopy Analysis technique is used. The findings of this work revealed that the magnetic parameter improves the heat transfer rate. This work also ensures that there are non-unique solutions of modeled problem for shrinking case and a unique solution for stretching case. Higher values of ${\text{Re}}_{x}$ results in declining of flow field. Rise in $M$ agrees to a decline in velocity distributions. Higher values of second order fluid parameter reduces the viscosity of fluid and accordingly velocity increases. Velocity profile is also a decreasing function of volume friction.

## Introduction

Nanofluid receives an enormous amount of attention over the last two decades by the researchers because of its high thermal conductivity and novel applications in different branches of science, engineering and technology. The conventional liquids consume low thermal conductivity thus it becomes inadequate for several heat transfer issues. The study of nanofluid is very important for the reason of its unique application that enhances the transfer of heat. That’s why scientists take interest to use nanofluid instead of regular fluids. Nanofluid shows a vital role at manufacturing level such as production of foods, electronics, biomedicines, transportations and cooling of nuclear reactors. Nanoparticles are very small in size (1–100 nm). The structure of nanoparticles contains a metal oxide, nitride, carbide and carbon tubes (SWCNTs and MWCNTs) etc. The quantity of nanopraticles in a base fluid was introduced first by Choi^1^ for increasing the thermal characteristics of such fluids. These fluids with heightened thermophysical properties were named as nanofluids. There are different types of nanofluids like fermium oxide, grapheneoxide, and carbon nanotubes etc. which are discussed in the literature. In the setting of medical flows, Akbar et al.^2^ utilized Buonjiornio’s model to explore systematically the peristaltic hydrodynamics of nanoliquids with wall slip impacts. Sayed et al.^3^ studied the influence of thermal transportation and alternative current on the peristaltic flow of a viscous dielectric fluid. Nakhchi and Esfahani^4^ examined a mathematical investigation for Cu–water nanoliquid flow through a spherical cylinder implanted with cross-cut warped tape with an alternative axis (CCTA). Furthermore, the reader can study about nanofluid in Refs.^5–7^.

Carbon nanotubes (CNTs) are round and hollow formed cylinders with indispensable qualities like great thermal conductivity and huge power makes them exceptionally appealing constituents in fluctuated applications for instance enhancer, drug delivery, optics and semiconductors etc. CNTs can be single or multi wall. Homogeneous carbon nanotube/polymer composites utilizing non-covalently functionalized, solvent single-walled carbon nanotubes (SWNTs) were created by Ramasubramaniam et al.^8^. Xue^9^ offered the carbon nanotubes (CNTs) alignment dissemination a new model of active thermal conductivity of CNTs. The study of SWCNT on peristaltic transportation of nanofluid in an inclined cylinder with penetrable walls is conferred by Nadeem et al.^10^. Homogeneous-heterogeneous reactions in the 3D flow of water-based nanoliquid soaking a permeable medium are demonstrated by Hayat et al.^11^. Nadeem et al.^12^ observed the transfer of heat by the influence of SWNCT and MWNCT with the state of oscillation. Late examinations featuring nanofluid impacts in different situations might be found in Ref.^13,14^. Various mathematical models have been used by the researcher over the linear stretched surfaces while limited work has been carried out in nonlinear or quadratic stretching.

The fluid of second grade is actually a subclass of non-Newtonian fluid for which flow field has a relationship up to second order derivative in terms of stress strain tensor. On the other hand, this relationship is of first order in case of the Newtonian fluids. Due to its importance, many researchers have diverted their attention towards the flow of second grade fluid. The study of thermal flow regarding this type of fluid is of more importance for researchers these days. The second grade liquid flow past a quadratic stretched surface analyzed by Cortell^15,16^. This idea further comprehended by Mahapatra and Sidui^17^. Gul et al.^18^ considered steady, axisymmetric and incompressible hybrid Nano liquid flow over an unending impermeable gyrating disk affected by a magnetic field, which has a few engineering and industrial applications. Sheikholeslami et al.^19^ discovered unsteady squeezing liquid flow amid corresponding surfaces. They reported that heat transfer escalates for nanoparticle concentration. Jawad et al.^20^ have deliberated the impression of variable thermal radiation over the unsteady 3-D flow of (SWCNTs) with water-base solutions.

A unique type of nanofluid that formed small metallic particles is called hybrid nanofluid. Hybrid nanofluid shares great applications in the field of engineering, agriculture, biological and applied sciences. Hybrid nanofluid increases the thermal efficiency at a very low cost. The electromagnetic radiation caused by the thermal flow of particles in the matter is termed as thermal radiation. These types of radiations are emitted by those matters which have a temperature higher than absolute zero. The motion of particles results in a charge acceleration that causes electromagnetic radiations. The use of hybrid nanofluid is more fruitful because their applications involve diesel engine oil, hybrid power engines, chillers improvement etc. Due to the importance of this class of fluid many researchers have carried out numerous investigations in this area by using different flow conditions and geometries.The exploratory works by Turcu et al.^21^ were the formerly contemplates that using the hybrid nanoparticles. Suresh et al.^22,23^ led the exploratory effort utilizing $Al_{2} O_{3} - Cu$ to examine the upgrade of the liquid thermal conductivity. Afterwards numerous investigators carried various studies for hybrid nanofluid by considering stretching/shrinking surfaces such as Waini et al.^24,25^, Zainal et al.^26^ and Khashi’ie et al.^27,28^. Relationships of thermal conductivity for ordinary nanofluids are completely examined and this study emphasizes planning, complications and challenges of hybrid nanofluids by Das^29^. Free convection of nanofluid in an inclined open cavity with a heat producing strong component is contemplated by Miroshnichenko et al.^30^. Siavashi and Rostami^31^ mathematically inspected the natural convection heat move of non-Newtonian water-nanoliquid inside a tube shaped annulus with a concentric round heat source secured with a conductive permeable layer. Huminic and Huminic^32^ presented a research review of the current results used in different heat exchangers, concerning the thermophysical properties and the characteristics flow of the heat transfer in hybrid nanofluids. The review denotes that the hybrid nanofluids may significantly increase the performance of heat exchangers. However, lots of research efforts are still required considering the hybrid nanoparticles combinations, the exact mixing ratio and its stability. Dinarv^33^ have presented torpor point limit layer flow of CuO–Ag/water hybrid nanoliquid. Dinarvand and Rostami^34^ have calculated systematically the incompressible laminar steady 3-D limit layer flow of a watery hybrid nanoliquid over an impervious turning plate with the steady spiral extending rate. Tayebi and Chamkha^35^ mathematically reviewed the heat transmission in an annulus between two confocal elliptic squares loaded up with hybrid $Al_{2} O_{3} - Cu$/water nanoliquid. A completely evolved laminar convective heat transfer and weight drop attributes through a consistently heated round cylinder utilizing $Al_{2} O_{3} - Cu$/water hybrid nanoliquid was introduced by Suresh et al.^36^. Jena et al.^37^ explained the combination of nano-composites utilizing hydrogen reduction methods from synthetically articulated mixtures. Volume concentration and temperature on ethylene glycol and MWCNT with dynamics viscosity of hybrid nanofluids was achieved by Afshari et al.^38^. `Unsteady radiative ethylene glycol-based carbon nanotube flow between two revolving disks was inspected by Ramzan et al.^39^. They marked that radial velocity and temperature gets increased for nanoparticle concentration. Barnoon et al.^40^ discussed rate of heat transmission with fluid flow using swirling circular obstacles. Afrand et al.^41^ have investigated 3-D free convective flow through vertically cylindrical annulus using molten gallium. In this work the authors have used horizontal magnetic field. Barnoon et al.^42^ have also discussed mixed convection with production of entropy using a square cavity where the cavity was filled with nanoparticles in the occurrence of magnetic effects. Farzinpour et al.^43^ have discussed the simulation for molecular dynamics using effects of ferro-nanofluid and unsteady magnetic field effects. Mousavi et al.^44^ have utilized the Lattice Boltzman method to explore the simulation of droplet detachment under the impact of electric field. Turkyilmazoglu et al.^45–48^ have carried out a tremendous work for heat transfer using nanofluid with different flow geometries and various flow conditions. Shah et al.^49^ have investigated the importance of suction and dual stretching on the motion of various types of nanofluids. Sheikholeslami et al.^50,51^ have carried out a tremendous work for heat transmission of nanofluid. The reader can further study about heat transfer through nanofluid flow in Refs.^52–56^.

The force exhibited by a particle due to magnetic and electric fields is termed as the Lorentz force. The force exerted on a particle due to the electric field is electric force and is given by the magnitude $F = qE$. On the other hand, a charged particle in a magnetic field will experience a force because of the magnetic field and is termed as a magnetic force where the particles are moving in a direction relative to the field motion. The combination of these two forces is termed as Lorentz force. Koriko et al.^57^ have discussed the significance of using $Fe_{3} O_{4}$ nanoparticles to alumina-water nanofluid past a stretching surface using Lorentz force. Vaidya et al.^58^ have studied the MHD peristaltic flow for non-Newtonian fluid through a narrowing asymmetric channel. The transportation of fluid using variable transport properties carried out over a porous surface. Aly and Pop^59^ have carried out the MHD flow of hybrid nanofluid flow past a shrinking/ stretching surface with a determination of double solution.

Most of the available literature is concern with the dispersion of the solid nanoparticles in the Newtonian fluids and very less literature available regarding the solid nano particles dispersion in the non-Newtonian fluids. In the current study we have focused the theoretical study of the non-Newtonian fluid considering the second grade fluid for the stable dispersion of the solid nanoparticles of $Al_{2} O_{3} - Cu$.

The newness of the present work is highlighted in the following points.

- Second-grade fluid used as a base fluid.
- $Al_{2} O_{3} - Cu$ are used as the solid nanoparticles.
- Magnetic field imposed vertically the flow pattern.
- Thermal radiation is included in the energy equation.
- The surface is nonlinearly stretching/shrinking.

## Mathematical formulation

**Figure 1:**
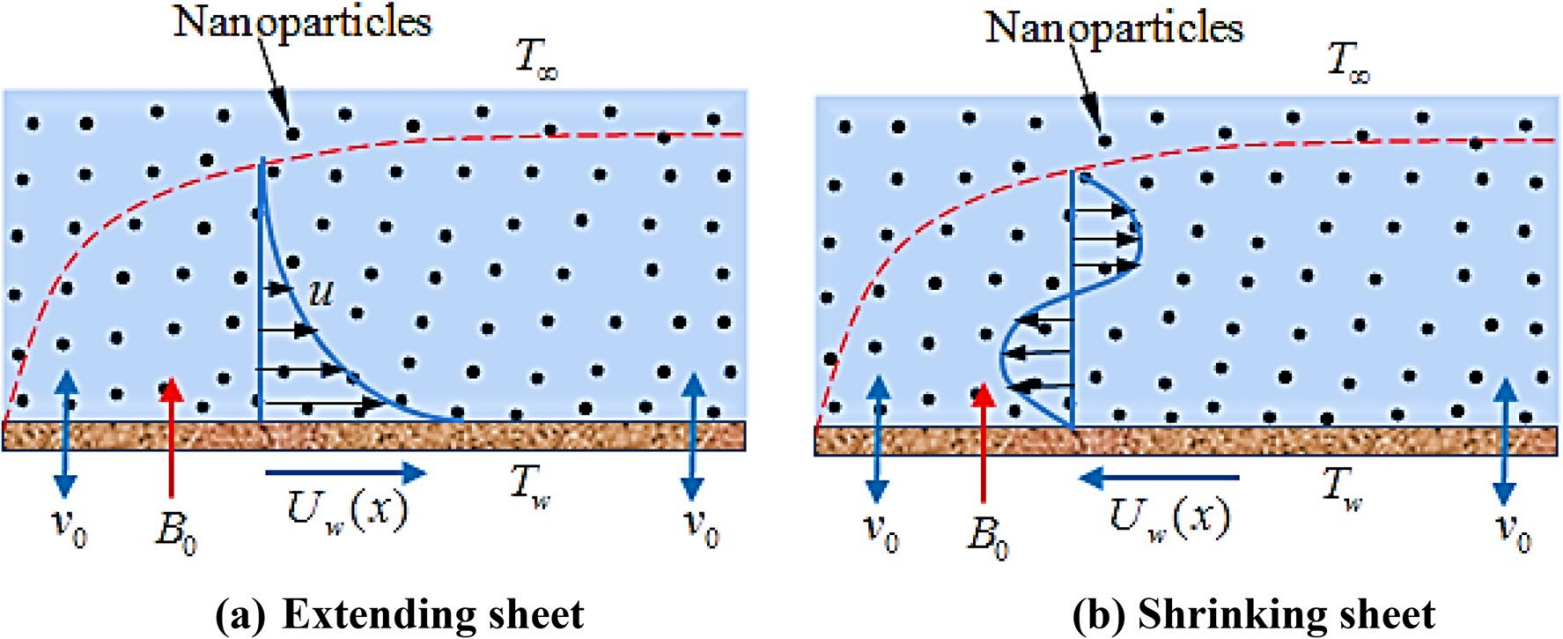
Physical sketch of the flow.

We assume here the steady MHD flow of an incompressible, electrically conducting Al2O3-Cu hybrid nanofluid towards a two-dimensional extending/shrinking sheet. Let $x$-direction is along the surface and $y$-axis being normal to the sheet along which magnetic field $\left( {B_{0} } \right)$ is applied. The schematic diagram is depicted in Fig. 1.

The flow is likely to be formed by a quadratic (non-linearly) extending/contracting sheet, which are employed along $x$-axis and in this way sheet velocity is $u_{w} \left( x \right) = ax + bx^{2}$. By connecting with the assessments of the common limit layer, we can compose the hybrid nanofluid governing equations^15–17,55,56^:

1 $$u_{x} + v_{y} = 0,$$

2 $$uu_{x} + vu_{y} = \upsilon_{hnf} u_{yy} + \alpha_{1} \left( {u_{x} u_{yy} + uu_{xyy} + u_{y} v_{yy} + vu_{yyy} } \right){ - }\frac{{\upsigma _{hnf} B_{0}^{2} u}}{{\rho_{hnf} }},$$

3 $$uT_{x} + vT_{y} = \frac{{k_{hnf} }}{{\left( {\rho c_{p} } \right)_{hnf} }}T_{yy} + \frac{{16\sigma^{*} }}{{\left( {\rho c_{p} } \right)_{hnf} k^{*} }}\left[ {{\rm T}^{3} {\rm T}_{yy} + 3{\rm T}^{2} \left( {T_{z} } \right)^{2} } \right],$$

Together with the boundary conditions^15–17,55,56^:

4 $$\begin{array}{*{20}l} {u = u_{w} \left( x \right) = \left( {ax + bx^{2} } \right)\lambda ,} \hfill & {v = v_{w} \left( x \right),T = T_{w} \,at\,y = 0,} \hfill \\ {u \to 0,T \to T_{\infty } ,} \hfill & {at\,\,y \to \infty .} \hfill \\ \end{array}$$

Above $u,\,v$ are the components of flow in $x,\,y$ directions,$\lambda$ is constant parameter with $\lambda > 0$ signifies that sheet is stretching while $\lambda < 0$ is a shrinking sheet. The value of $T_{w}$ is depicted as $T_{w} \left( x \right) = T_{\infty } + T_{\infty } \left( {\frac{x}{{L^{2} }}} \right)$. The thermophoresis characteristics of alumina $\left( {Al_{2} O_{3} } \right)$ and copper $\left( {Cu} \right)$ are given in Table 1.

**Table 1:** Numerical values of thermophysical properties of nanoparticles and pure fluid^26^.

Physical properties	$\left( {Al_{2} O_{3} } \right)$	$\left( {Cu} \right)$
$\rho \,\left( {{\text{kg}}/{\text{m}}^{3} } \right)$	3970	8933
$B \times 10^{ - 5} \,\left( {{\text{mK}}} \right)$	0.85	1.67
$c_{p} \,\left( {{\text{J}}/{\text{kgK}}} \right)$	765	385
$k\,\left( {{\text{W}}/{\text{mK}}} \right)$	40	400

Thermophysical properties of hybrid nanofluids are shown in Eq. (5)^26^:

5 $$\begin{aligned} & \left( {\rho C_{p} } \right)_{hnf} = \left( {1 - \phi_{2} } \right)\left[ {\left( {1 - \phi_{1} } \right)\left( {\rho C_{p} } \right)_{f} + \phi_{1} \left( {\rho C_{p} } \right)_{{s_{1} }} } \right] \\ & \mu_{hnf} = \frac{{\mu_{f} }}{{\left( {1 - \phi_{1} } \right)^{2.5} \left( {1 - \phi_{2} } \right)^{2.5} }} \\ & \frac{{k_{hnf} }}{{k_{nf} }} = \frac{{k_{{s_{2} }} + 2k_{nf} - 2\phi_{2} \left( {k_{nf} - k_{{s_{2} }} } \right)}}{{k_{{s_{2} }} + 2k_{nf} + \phi_{2} \left( {k_{nf} - k_{{s_{2} }} } \right)}},\frac{{k_{nf} }}{{k_{f} }} = \frac{{k_{{s_{1} }} + 2k_{f} - 2\phi_{{s_{1} }} (k_{f} - k_{{s_{1} }} )}}{{k_{{s_{1} }} + 2k_{f} + \phi_{{s_{1} }} (k_{f} - k_{{s_{1} }} )}}, \\ & \frac{{\sigma_{hnf} }}{{\sigma_{bf} }} = \left[ {\frac{{\left( {\sigma_{{s_{2} }} - \sigma_{bf} } \right)3\phi_{{s_{2} }} }}{{\left( {\sigma_{{s_{2} }} + 2\sigma_{bf} } \right) + \left( {\sigma_{bf} - \sigma_{{s_{2} }} } \right)\phi_{{s_{2} }} }} + 1} \right],\frac{{\sigma_{bf} }}{{\sigma_{f} }} = \left[ {\frac{{\left( {\sigma_{f} - \sigma_{{s_{1} }} } \right)3\phi_{{s_{1} }} }}{{\left( {\sigma_{{s_{1} }} - \sigma_{f} } \right) - \left( {\sigma_{{s_{1} }} + 2\sigma_{f} } \right)}} + 1} \right], \\ & \rho_{hnf} = \left( {1 - \phi_{2} } \right)\left[ {\left( {1 - \phi_{1} } \right)\left( \rho \right)_{f} + \phi_{1} \left( \rho \right)_{{s_{1} }} } \right] + \phi_{2} \left( \rho \right)_{{s_{2} }} . \\ \end{aligned}$$

Considering Takhar et al.^44^, the group of similar variables is defined as:

6 $$u = axf^{\prime}\left( \eta \right) + bx^{2} g^{\prime}\left( \eta \right),v = - \sqrt {a\nu_{f} } f\left( \eta \right) - \frac{2bx}{{\sqrt {\frac{a}{{\nu_{f} }}} }}g\left( \eta \right),\theta \left( \eta \right) = \frac{{T - T_{\infty } }}{{T_{w} - T_{\infty } }},\eta = y\sqrt {\frac{a}{{\nu_{f} }}} ,$$

Hence that:

7 $$v_{w} = - \sqrt {a\nu_{f} } S_{1} - \frac{2bx}{{\sqrt {\frac{a}{{\nu_{f} }}} }}S_{2}$$

Here, $S_{1} ,S_{2}$ are the transpiration parameters with $\left( {S_{1} ,S_{2} } \right) > 0$ for suction and $\left( {S_{1} ,S_{2} } \right) < 0$ for blowing and injection constraint. By using the Eq. (5), Eqs. (2, 3) are changed to:

8 $$\begin{aligned} & f^{\prime\prime\prime} - \left( {1 - \phi_{1} } \right)^{2.5} \left( {1 - \phi_{2} } \right)^{2.5} \left( {1 - \phi_{2} } \right)\left[ {\left[ {\left( {1 - \phi_{1} } \right)\left( \rho \right)_{f} + \phi_{1} \left( \rho \right)_{{s_{1} }} } \right] + \phi_{2} \left( \rho \right)_{{s_{2} }} } \right]\\&\quad\beta \left( {2f^{\prime\prime\prime}f^{\prime} - ff^{iv} - f^{{\prime\prime}{2}} } \right) - ff^{\prime\prime} + f^{{\prime}{2}} - \left( {1 - \phi_{1} } \right)^{2.5} \left( {1 - \phi_{2} } \right)^{2.5} \frac{{\sigma_{hnf} }}{{\sigma_{f} }}Mf^{\prime} = 0, \end{aligned}$$

9 $$\begin{aligned} & g^{\prime\prime\prime} - fg^{\prime\prime} + 3f^{\prime}g^{\prime} - 2gf^{\prime\prime} - \left( {1 - \phi_{1} } \right)^{2.5} \left( {1 - \phi_{2} } \right)^{2.5} \left( {1 - \phi_{2} } \right)\left[ {\left[ {\left( {1 - \phi_{1} } \right)\left( \rho \right)_{f} + \phi_{1} \left( \rho \right)_{{s_{1} }} } \right] + \phi_{2} \left( \rho \right)_{{s_{2} }} } \right]\\ &\quad\beta \left( {3f^{\prime}g^{\prime\prime\prime} + 3f^{\prime\prime\prime}g^{\prime} - 2gf^{iv} - 3f^{\prime\prime}g^{\prime\prime} - fg^{iv} } \right) - \left( {1 - \phi_{1} } \right)^{2.5} \left( {1 - \phi_{2} } \right)^{2.5} \frac{{\sigma_{hnf} }}{{\sigma_{f} }}Mg^{\prime} = 0, \end{aligned}$$

10 $$\left( {g^{\prime}} \right)^{2} - gg^{\prime\prime} + \left( {1 - \phi_{1} } \right)^{2.5} \left( {1 - \phi_{2} } \right)^{2.5} \left( {1 - \phi_{2} } \right)\left[ {\left[ {\left( {1 - \phi_{1} } \right)\left( \rho \right)_{f} + \phi_{1} \left( \rho \right)_{{s_{1} }} } \right] + \phi_{2} \left( \rho \right)_{{s_{2} }} } \right]\beta \left( {\left( {g^{\prime\prime}} \right)^{2} + gg^{(iv)} - 2g^{\prime}g^{\prime\prime\prime}} \right) = 0,$$

11 $$\frac{{\frac{{k_{hnf} }}{{k_{f} }}}}{{\frac{{\left( {\rho cp} \right)_{hnf} }}{{\left( {\rho cp} \right)_{f} }}}}\left( {\theta^{\prime\prime} + R\left[ {\left( {1 + \left( {\theta_{w} - 1} \right)\theta } \right)^{3} \theta^{\prime\prime}} \right]} \right) + \Pr \left( {f + k_{1} g} \right)\theta^{\prime} = 0,$$

Subjected to the conditions:

12 $$\begin{aligned} & f\left( 0 \right) = S_{1} ,g\left( 0 \right) = S_{2} ,f^{\prime}\left( 0 \right) = \lambda ,g^{\prime}\left( 0 \right) = \lambda ,\theta \left( 0 \right) = 1, \\ & f^{\prime}\left( \infty \right) \to 0,g^{\prime}\left( \infty \right) \to 0,\theta \left( \infty \right) \to 0. \\ \end{aligned}$$

13 $$M = \frac{{\sigma_{f} B_{0}^{2} }}{{a\rho_{f} }},\Pr = \frac{{\nu_{f} }}{{\alpha_{f} }},R = \frac{{16\sigma^{*} {\rm T}_{\infty }^{3} }}{{3k_{nf} k^{*} }},\theta_{w} = \frac{{{\rm T}_{w} }}{{{\rm T}_{\alpha } }},\beta_{1} = \frac{{\alpha_{1} }}{{\rho_{f} ax^{2} }}.$$

Above the symbols $M,\beta ,\Pr ,R\,,\theta_{w} \,and\,k_{1} = \frac{cx}{a}$ respectively denote magnetic parameter, second grade fluid parameter, Prandtl number, radiation parameter, temperature ratio parameter and nonlinear stretching parameter.

### Quantities of engineering interest

The engineering quantities of interest are $C{}_{fx}$ and $Nu_{x}$ that is denoted as^16^;

14 $$C_{fx} = \frac{{\tau_{w} }}{{\rho \left( {ax} \right)^{2} }},Nu_{x} = \frac{{xq_{w} }}{{k_{f} \left( {T_{w} - T_{\infty } } \right)}},$$

The surface heat flux $q_{w}$ and $\tau_{w}$ are written as;

15 $$\tau_{w} = \frac{{\mu_{hnf} }}{{\rho_{f} }}\left( {u_{y} } \right)_{y = 0} ,q_{w} = - k_{hnf} \left( {T_{y} } \right)_{y = 0} + \left( {qr} \right)_{w} ,$$

By the use of Eqs. (13) and (14), then we have;

16 $$C_{fx} Re_{x}^{\frac{1}{2}} = \frac{{\mu_{hnf} }}{{\mu_{nf} }}\left[ {f^{\prime\prime}\left( 0 \right) + \beta_{1} xg^{\prime\prime}\left( 0 \right)} \right],Nu_{x} Re_{x}^{{ - \frac{1}{2}}} = - \frac{{k_{hnf} }}{{k_{f} }}(1 + R\theta_{w}^{3} )\theta^{\prime}\left( 0 \right).$$

Above $Re_{x} = \frac{{\upsilon_{f} }}{{ax^{2} }}$ depicts local Reynolds number while $\beta_{1}$ is a dimension free parameter with $\beta_{1} = \frac{b}{a}$.

## Solution by HAM

The problem is explained through the HAM^60–63^ method. The detail of HAM for current modeled problem is describes as follows:

17 $$L_{{\overset{\lower0.5em\hbox{$\smash{\scriptscriptstyle\frown}$}}{f} }} (\overset{\lower0.5em\hbox{$\smash{\scriptscriptstyle\frown}$}}{f} ) = \overset{\lower0.5em\hbox{$\smash{\scriptscriptstyle\frown}$}}{f}^{\prime\prime\prime},{\text{L}}_{{\overset{\lower0.5em\hbox{$\smash{\scriptscriptstyle\frown}$}}{\theta } }} {(}\overset{\lower0.5em\hbox{$\smash{\scriptscriptstyle\frown}$}}{\theta } {) = }\overset{\lower0.5em\hbox{$\smash{\scriptscriptstyle\frown}$}}{\theta }^{\prime\prime}{, }$$

Linear operators $$L_{{\overset{\lower0.5em\hbox{$\smash{\scriptscriptstyle\frown}$}}{f} }} ,\,and\,{\text{L}}_{{\overset{\lower0.5em\hbox{$\smash{\scriptscriptstyle\frown}$}}{\theta } }}$$ are signified as

18 $$L_{{\overset{\lower0.5em\hbox{$\smash{\scriptscriptstyle\frown}$}}{f} }} (e_{1} + e_{2} \eta + e_{3} \eta^{2} ) = 0,{\text{L}}_{{\overset{\lower0.5em\hbox{$\smash{\scriptscriptstyle\frown}$}}{\theta } }} (e_{4} + e_{5} \eta ) = 0, \,$$

The constant non-linear operators are $${\rm N}_{{\overset{\lower0.5em\hbox{$\smash{\scriptscriptstyle\frown}$}}{f} }} \,and\,\,{\rm N}_{{\overset{\lower0.5em\hbox{$\smash{\scriptscriptstyle\frown}$}}{\theta } }}$$ with

19 $$\begin{aligned} {\rm N}_{{\overset{\lower0.5em\hbox{$\smash{\scriptscriptstyle\frown}$}}{f} }} \, \left[ {\overset{\lower0.5em\hbox{$\smash{\scriptscriptstyle\frown}$}}{f} (\eta ;\zeta )} \right] &= \overset{\lower0.5em\hbox{$\smash{\scriptscriptstyle\frown}$}}{f}_{\eta \eta \eta } - \left( {1 - \phi_{1} } \right)^{2.5} \left( {1 - \phi_{2} } \right)^{2.5} \left( {1 - \phi_{2} } \right)\left[ {\left[ {\left( {1 - \phi_{1} } \right)\left( \rho \right)_{f} + \phi_{1} \left( \rho \right)_{{s_{1} }} } \right] + \phi_{2} \left( \rho \right)_{{s_{2} }} } \right]\\&\quad\beta \left( {2\overset{\lower0.5em\hbox{$\smash{\scriptscriptstyle\frown}$}}{f}_{\eta } \overset{\lower0.5em\hbox{$\smash{\scriptscriptstyle\frown}$}}{f}_{\eta \eta \eta \eta } + \overset{\lower0.5em\hbox{$\smash{\scriptscriptstyle\frown}$}}{f}_{{^{\eta \eta } }}^{2} - \overset{\lower0.5em\hbox{$\smash{\scriptscriptstyle\frown}$}}{f} \overset{\lower0.5em\hbox{$\smash{\scriptscriptstyle\frown}$}}{f}_{\eta \eta \eta \eta } } \right) - \overset{\lower0.5em\hbox{$\smash{\scriptscriptstyle\frown}$}}{f} \overset{\lower0.5em\hbox{$\smash{\scriptscriptstyle\frown}$}}{f}_{\eta \eta } + \left( {\overset{\lower0.5em\hbox{$\smash{\scriptscriptstyle\frown}$}}{f}_{\eta } } \right)^{2} - \left( {1 - \phi_{1} } \right)^{2.5} \left( {1 - \phi_{2} } \right)^{2.5} \frac{{\sigma_{hnf} }}{{\sigma_{f} }}M\overset{\lower0.5em\hbox{$\smash{\scriptscriptstyle\frown}$}}{f}_{\eta } \end{aligned}$$

20 $$\begin{aligned} {\rm N}_{{\overset{\lower0.5em\hbox{$\smash{\scriptscriptstyle\frown}$}}{g} }} \left[ {\overset{\lower0.5em\hbox{$\smash{\scriptscriptstyle\frown}$}}{g} (\eta ;\zeta )} \right] &= \overset{\lower0.5em\hbox{$\smash{\scriptscriptstyle\frown}$}}{g}_{\eta \eta \eta } - \left( {1 - \phi_{1} } \right)^{2.5} \left( {1 - \phi_{2} } \right)^{2.5} \left( {1 - \phi_{2} } \right)\left[ {\left[ {\left( {1 - \phi_{1} } \right)\left( \rho \right)_{f} + \phi_{1} \left( \rho \right)_{{s_{1} }} } \right] + \phi_{2} \left( \rho \right)_{{s_{2} }} } \right]\\ &\quad\beta \left( {3\overset{\lower0.5em\hbox{$\smash{\scriptscriptstyle\frown}$}}{f}_{\eta } \overset{\lower0.5em\hbox{$\smash{\scriptscriptstyle\frown}$}}{g}_{\eta \eta \eta } + 3\overset{\lower0.5em\hbox{$\smash{\scriptscriptstyle\frown}$}}{f}_{\eta \eta \eta } \overset{\lower0.5em\hbox{$\smash{\scriptscriptstyle\frown}$}}{g}_{\eta } - \overset{\lower0.5em\hbox{$\smash{\scriptscriptstyle\frown}$}}{f} \overset{\lower0.5em\hbox{$\smash{\scriptscriptstyle\frown}$}}{g}_{\eta \eta \eta \eta } - 3\overset{\lower0.5em\hbox{$\smash{\scriptscriptstyle\frown}$}}{f}_{\eta \eta } \overset{\lower0.5em\hbox{$\smash{\scriptscriptstyle\frown}$}}{g}_{\eta \eta } - 2\overset{\lower0.5em\hbox{$\smash{\scriptscriptstyle\frown}$}}{f}_{\eta \eta \eta \eta } \overset{\lower0.5em\hbox{$\smash{\scriptscriptstyle\frown}$}}{g} } \right) - \overset{\lower0.5em\hbox{$\smash{\scriptscriptstyle\frown}$}}{f} \overset{\lower0.5em\hbox{$\smash{\scriptscriptstyle\frown}$}}{g}_{\eta \eta } + 3\overset{\lower0.5em\hbox{$\smash{\scriptscriptstyle\frown}$}}{f}_{\eta } \overset{\lower0.5em\hbox{$\smash{\scriptscriptstyle\frown}$}}{g}_{\eta } - 2\overset{\lower0.5em\hbox{$\smash{\scriptscriptstyle\frown}$}}{f}_{\eta \eta } \overset{\lower0.5em\hbox{$\smash{\scriptscriptstyle\frown}$}}{g} \\ &\\ &\quad- \left( {1 - \phi_{1} } \right)^{2.5} \left( {1 - \phi_{2} } \right)^{2.5} \frac{{\sigma_{hnf} }}{{\sigma_{f} }}M\overset{\lower0.5em\hbox{$\smash{\scriptscriptstyle\frown}$}}{g}_{\eta } , \end{aligned}$$

21 $${\rm N}_{{\overset{\lower0.5em\hbox{$\smash{\scriptscriptstyle\frown}$}}{\theta } }} \left[ {\overset{\lower0.5em\hbox{$\smash{\scriptscriptstyle\frown}$}}{f} (\eta ;\zeta ),\overset{\lower0.5em\hbox{$\smash{\scriptscriptstyle\frown}$}}{\theta } (\eta ;\zeta ),} \right] = \frac{{\frac{{k_{hnf} }}{{k_{f} }}}}{{\frac{{\left( {\rho cp} \right)_{hnf} }}{{\left( {\rho cp} \right)_{f} }}}}\left[ {\frac{1}{\Pr }\overset{\lower0.5em\hbox{$\smash{\scriptscriptstyle\frown}$}}{\theta }_{\eta \eta } + R\left( {1 + \left( {\theta_{w} - 1} \right)\overset{\lower0.5em\hbox{$\smash{\scriptscriptstyle\frown}$}}{\theta } } \right)^{3} \overset{\lower0.5em\hbox{$\smash{\scriptscriptstyle\frown}$}}{\theta }_{\eta \eta } + 3\left( {1 + \left( {\theta_{w} - 1} \right)\overset{\lower0.5em\hbox{$\smash{\scriptscriptstyle\frown}$}}{\theta } } \right)^{2} \left( {\theta_{w} - 1} \right)\overset{\lower0.5em\hbox{$\smash{\scriptscriptstyle\frown}$}}{\theta }_{{^{\eta } }}^{2} } \right] + \left( {f + g} \right)\overset{\lower0.5em\hbox{$\smash{\scriptscriptstyle\frown}$}}{\theta }_{\eta } ,$$

For Eqs. (8–10) the 0th-order system is written as

22 $$(1 - \eta )L_{{\overset{\lower0.5em\hbox{$\smash{\scriptscriptstyle\frown}$}}{f} }} \left[ {\overset{\lower0.5em\hbox{$\smash{\scriptscriptstyle\frown}$}}{f} (\eta ;\zeta ) - \overset{\lower0.5em\hbox{$\smash{\scriptscriptstyle\frown}$}}{f}_{0} (\eta )} \right] = p\hbar_{{\overset{\lower0.5em\hbox{$\smash{\scriptscriptstyle\frown}$}}{f} }} {\rm N}_{{\overset{\lower0.5em\hbox{$\smash{\scriptscriptstyle\frown}$}}{f} }} \, \left[ {\overset{\lower0.5em\hbox{$\smash{\scriptscriptstyle\frown}$}}{f} (\eta ;\zeta )} \right]$$

23 $$(1 - \eta )L_{{\overset{\lower0.5em\hbox{$\smash{\scriptscriptstyle\frown}$}}{g} }} \left[ {\overset{\lower0.5em\hbox{$\smash{\scriptscriptstyle\frown}$}}{g} (\eta ;\zeta ) - \overset{\lower0.5em\hbox{$\smash{\scriptscriptstyle\frown}$}}{g}_{0} (\eta )} \right] = p\hbar_{{\overset{\lower0.5em\hbox{$\smash{\scriptscriptstyle\frown}$}}{g} }} {\rm N}_{{\overset{\lower0.5em\hbox{$\smash{\scriptscriptstyle\frown}$}}{g} }} \, \left[ {\overset{\lower0.5em\hbox{$\smash{\scriptscriptstyle\frown}$}}{g} (\eta ;\zeta )} \right]$$

24 $$(1 - \eta ) \, L_{{\overset{\lower0.5em\hbox{$\smash{\scriptscriptstyle\frown}$}}{\theta } }} \left[ {\overset{\lower0.5em\hbox{$\smash{\scriptscriptstyle\frown}$}}{\theta } (\eta ;\zeta ) - \overset{\lower0.5em\hbox{$\smash{\scriptscriptstyle\frown}$}}{\theta }_{0} (\eta )} \right] = p\hbar_{{\overset{\lower0.5em\hbox{$\smash{\scriptscriptstyle\frown}$}}{\theta } }} {\rm N}_{{\overset{\lower0.5em\hbox{$\smash{\scriptscriptstyle\frown}$}}{\theta } }} \left[ {\overset{\lower0.5em\hbox{$\smash{\scriptscriptstyle\frown}$}}{f} (\eta ;\zeta ),\overset{\lower0.5em\hbox{$\smash{\scriptscriptstyle\frown}$}}{\theta } (\eta ;\zeta )} \right]$$

whereas BCs are:

25 $$\begin{aligned} & \left. {\overset{\lower0.5em\hbox{$\smash{\scriptscriptstyle\frown}$}}{f} (\eta ;\zeta )} \right|_{\eta = 0} = S_{1} ,\left. {\overset{\lower0.5em\hbox{$\smash{\scriptscriptstyle\frown}$}}{g} (\eta ;\zeta )} \right|_{\eta = 0} = S_{2} \, \left. {\frac{{\partial \overset{\lower0.5em\hbox{$\smash{\scriptscriptstyle\frown}$}}{f} (\eta ;\zeta )}}{\partial \eta }} \right|_{\eta = 0} = \lambda ,\left. {\frac{{\partial \overset{\lower0.5em\hbox{$\smash{\scriptscriptstyle\frown}$}}{g} (\eta ;\zeta )}}{\partial \eta }} \right|_{\eta = 0} = \lambda ,\left. {\overset{\lower0.5em\hbox{$\smash{\scriptscriptstyle\frown}$}}{\theta } (\eta ;\zeta )} \right|_{\eta = 0} = 1, \\ & \left. {\frac{{\partial \overset{\lower0.5em\hbox{$\smash{\scriptscriptstyle\frown}$}}{f} (\eta ;\zeta )}}{\partial \eta }} \right|_{\eta = \infty } = 0,\left. {\frac{{\partial \overset{\lower0.5em\hbox{$\smash{\scriptscriptstyle\frown}$}}{g} (\eta ;\zeta )}}{\partial \eta }} \right|_{\eta = \infty } = 0,\left. {\overset{\lower0.5em\hbox{$\smash{\scriptscriptstyle\frown}$}}{\theta } (\eta ;\zeta )} \right|_{\eta = \infty } = 0,\left. {\overset{\lower0.5em\hbox{$\smash{\scriptscriptstyle\frown}$}}{\phi } (\eta ;\zeta )} \right|_{\eta = \infty } = 0. \\ \end{aligned}$$

While the implanting constraint is $$\zeta \in [0,1][0,1]$$, to adjust for the solution convergence $$\hbar_{{\overset{\lower0.5em\hbox{$\smash{\scriptscriptstyle\frown}$}}{f} }}$$ , $$\hbar_{{\overset{\lower0.5em\hbox{$\smash{\scriptscriptstyle\frown}$}}{g} }}$$ and $$\hbar_{{\overset{\lower0.5em\hbox{$\smash{\scriptscriptstyle\frown}$}}{\theta } }}$$ are utilized. When $$\zeta = 0{\text{ and }}\zeta = 1$$ we have:

26 $$\overset{\lower0.5em\hbox{$\smash{\scriptscriptstyle\frown}$}}{f} (\eta ;1) = \overset{\lower0.5em\hbox{$\smash{\scriptscriptstyle\frown}$}}{f} (\eta ),\overset{\lower0.5em\hbox{$\smash{\scriptscriptstyle\frown}$}}{g} (\xi ;1) = \overset{\lower0.5em\hbox{$\smash{\scriptscriptstyle\frown}$}}{g} (\eta ),\overset{\lower0.5em\hbox{$\smash{\scriptscriptstyle\frown}$}}{\theta } (\eta ;1) = \overset{\lower0.5em\hbox{$\smash{\scriptscriptstyle\frown}$}}{\theta } (\eta ) \,$$

Enlarge the $$\overset{\lower0.5em\hbox{$\smash{\scriptscriptstyle\frown}$}}{f} (\eta ;\zeta ) \, ,\overset{\lower0.5em\hbox{$\smash{\scriptscriptstyle\frown}$}}{g} (\eta ;\zeta )$$ and $$\overset{\lower0.5em\hbox{$\smash{\scriptscriptstyle\frown}$}}{\theta } (\eta ;\zeta )$$ over Taylor’s series for $$\zeta = 0$$

27 $$\begin{aligned} \overset{\lower0.5em\hbox{$\smash{\scriptscriptstyle\frown}$}}{f} (\eta ;\zeta ) & = \, \overset{\lower0.5em\hbox{$\smash{\scriptscriptstyle\frown}$}}{f}_{0} (\eta ) + \sum\nolimits_{n = 1}^{\infty } {\overset{\lower0.5em\hbox{$\smash{\scriptscriptstyle\frown}$}}{f}_{n} (\eta )\zeta^{n} } \\ \overset{\lower0.5em\hbox{$\smash{\scriptscriptstyle\frown}$}}{g} (\eta ;\zeta ) & = \, \overset{\lower0.5em\hbox{$\smash{\scriptscriptstyle\frown}$}}{g}_{0} (\eta ) + \sum\nolimits_{n = 1}^{\infty } {\overset{\lower0.5em\hbox{$\smash{\scriptscriptstyle\frown}$}}{g}_{n} (\eta )\zeta^{n} } \\ \overset{\lower0.5em\hbox{$\smash{\scriptscriptstyle\frown}$}}{\theta } (\eta ;\zeta ) & = \, \overset{\lower0.5em\hbox{$\smash{\scriptscriptstyle\frown}$}}{\theta }_{0} (\eta ) + \sum\nolimits_{n = 1}^{\infty } {\overset{\lower0.5em\hbox{$\smash{\scriptscriptstyle\frown}$}}{\theta }_{n} (\eta )\zeta^{n} } \eta \\ \end{aligned}$$

28 $$\overset{\lower0.5em\hbox{$\smash{\scriptscriptstyle\frown}$}}{f}_{n} (\eta ) \, = \left. {\frac{1}{n!}\frac{{\partial \overset{\lower0.5em\hbox{$\smash{\scriptscriptstyle\frown}$}}{f} (\eta ;\zeta )}}{\partial \eta }} \right|_{p = 0} ,\overset{\lower0.5em\hbox{$\smash{\scriptscriptstyle\frown}$}}{g}_{n} (\eta ) \, = \left. {\frac{1}{n!}\frac{{\partial \overset{\lower0.5em\hbox{$\smash{\scriptscriptstyle\frown}$}}{g} (\eta ;\zeta )}}{\partial \eta }} \right|_{p = 0} \overset{\lower0.5em\hbox{$\smash{\scriptscriptstyle\frown}$}}{\theta }_{n} (\eta ) \, = \left. {\frac{1}{n!}\frac{{\partial \overset{\lower0.5em\hbox{$\smash{\scriptscriptstyle\frown}$}}{\theta } (\eta ;\zeta )}}{\partial \eta }} \right|_{p = 0} \, {. }$$

whereas BCs are:

29 $$\overset{\lower0.5em\hbox{$\smash{\scriptscriptstyle\frown}$}}{f} \left( 0 \right) = S_{1} ,\overset{\lower0.5em\hbox{$\smash{\scriptscriptstyle\frown}$}}{g} \left( 0 \right) = S_{2} ,\overset{\lower0.5em\hbox{$\smash{\scriptscriptstyle\frown}$}}{f^{\prime}} \left( 0 \right) = \lambda ,\overset{\lower0.5em\hbox{$\smash{\scriptscriptstyle\frown}$}}{g^{\prime}} \left( 0 \right) = \lambda ,\overset{\lower0.5em\hbox{$\smash{\scriptscriptstyle\frown}$}}{\theta } \left( 0 \right) = 1,\overset{\lower0.5em\hbox{$\smash{\scriptscriptstyle\frown}$}}{f^{\prime}} \left( \infty \right) = 0,\overset{\lower0.5em\hbox{$\smash{\scriptscriptstyle\frown}$}}{g}^{\prime}\left( \infty \right) = 0,\overset{\lower0.5em\hbox{$\smash{\scriptscriptstyle\frown}$}}{\theta } \left( \infty \right) = 0.$$

where

30 $${\text{While}}\,\chi_{n} = \left\{ {\begin{array}{*{20}l} {0,} \hfill & {\quad {\text{if}}\,n \le 1} \hfill \\ {1,} \hfill & {\quad {\text{if}}\,n > 1.} \hfill \\ \end{array} } \right.$$

## Results and discussions

**Figure 2:**
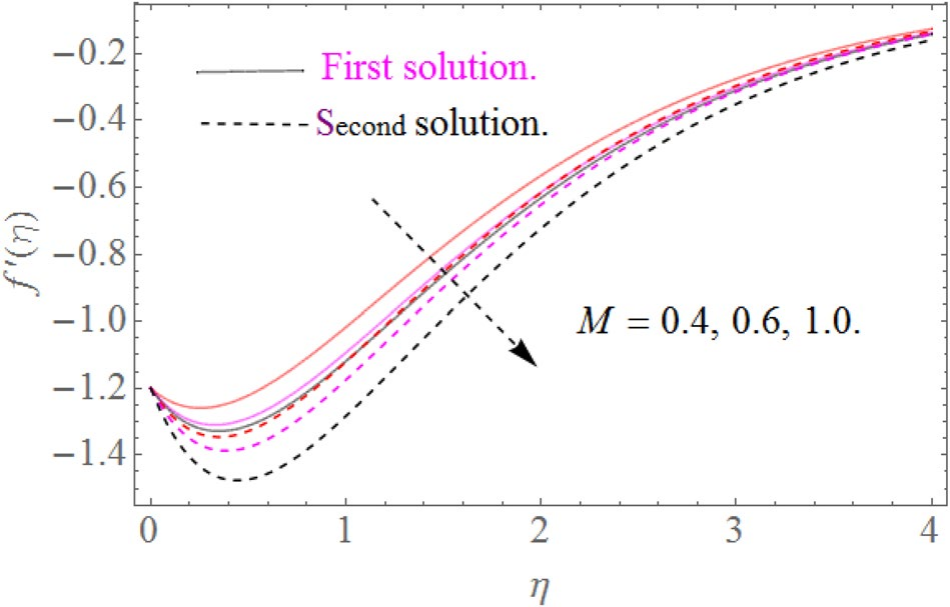
Influence of $M$ on $f^{\prime}(\eta )$ for $Re_{x} = 5,\beta = 0.2,\phi_{2} = 0.02$.

**Figure 3:**
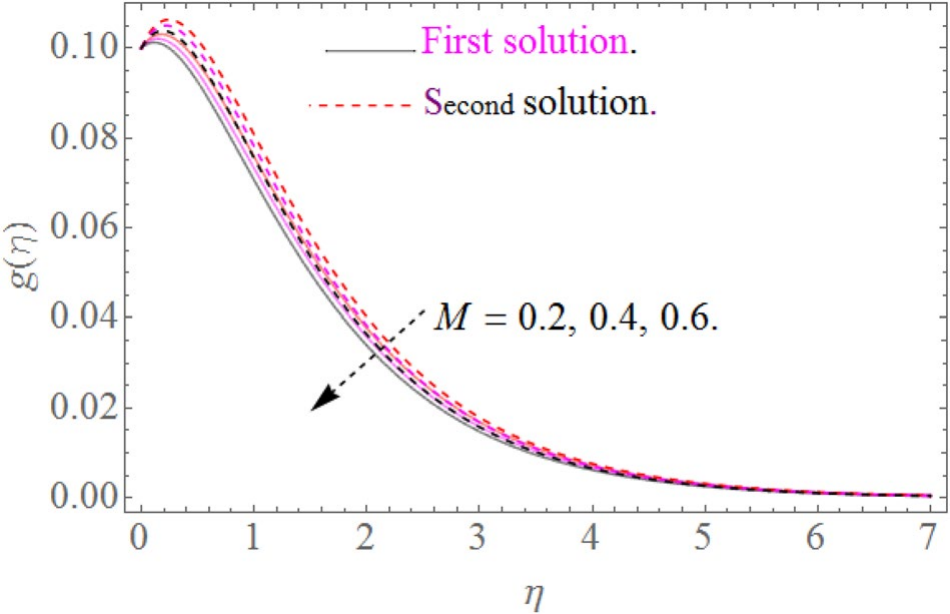
Influence of $M$ on $g(\eta )$ for $Re_{x} = 5,\beta = 0.2,\phi_{2} = 0.02$.

**Figure 4:**
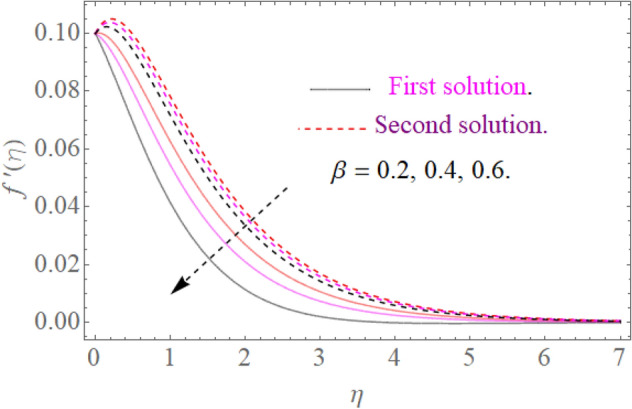
Influence of $\beta$ on $f^{\prime}(\eta )$ for $M = 0.9,\phi_{2} = 0.02$.

**Figure 5:**
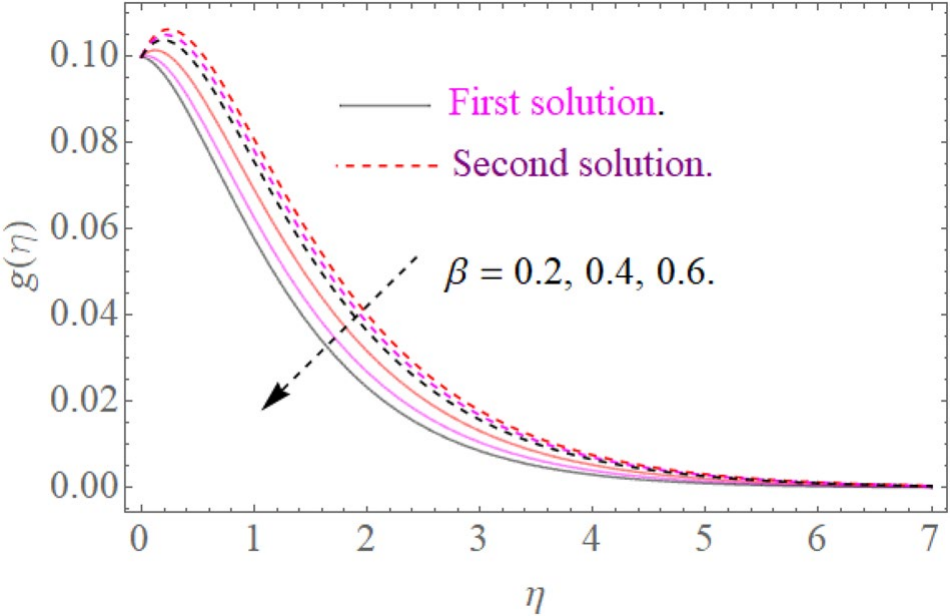
Influence of $\beta$ on $g(\eta )$ for $M = 0.9,\phi_{2} = 0.02$.

**Figure 6:**
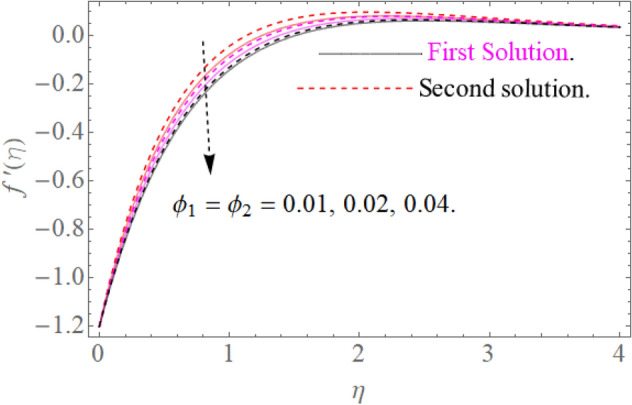
Influence of $\phi_{2}$ on $f^{\prime}(\eta )$ for $M = 0.9,\beta = 0.2$.

**Figure 7:**
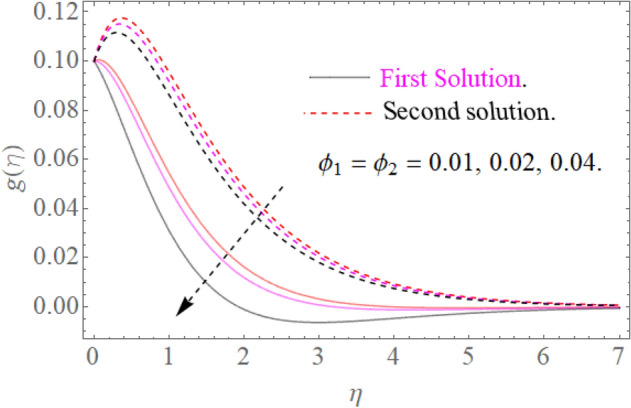
Influence of $\phi_{2}$ on $g(\eta )$ for $M = 0.9,\beta = 0.2$.

**Figure 8:**
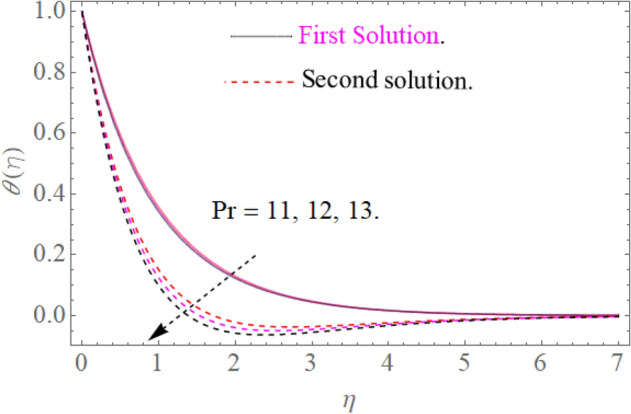
Influence of $\Pr$ on $\theta (\eta )$ for $R = 0.3,\theta_{w} = 1.2$.

**Figure 9:**
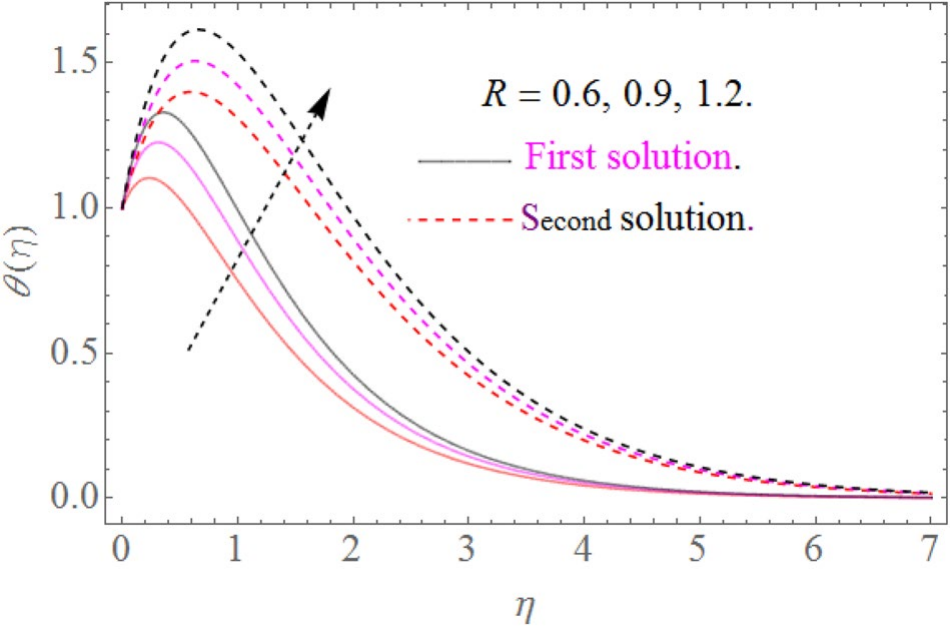
Influence of $R$ on $\theta (\eta )$ for $\Pr = 10.2,\theta_{w} = 1.2$.

**Figure 10:**
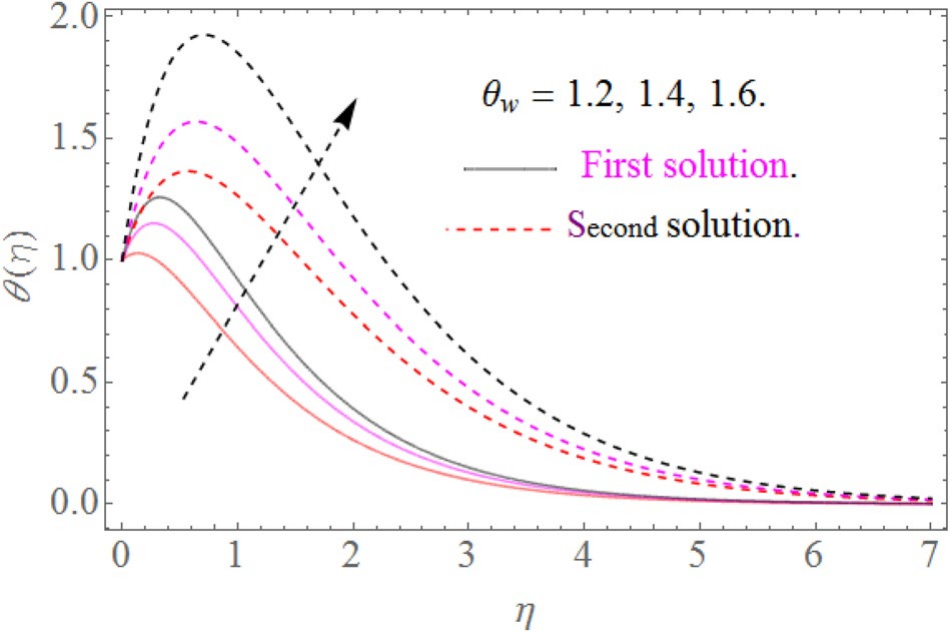
Impact of $\theta_{w}$ on $\theta (\eta )$ for $R = 0.3,\Pr = 10.2$.

**Figure 11:**
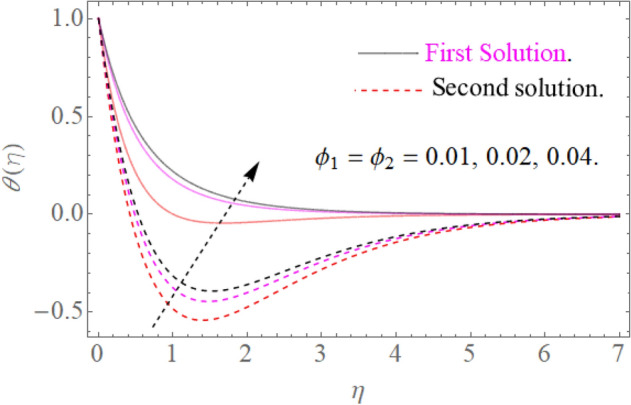
Influence of $\phi_{1} ,\phi_{2}$ on $\theta (\eta )$ for $R = 0.3,\Pr = 10.2$.

In this section, we now deliberate the different outcomes of the existing study and displayed graphically in Figs. 2, 3, 4, 5, 6, 7, 8, 9, 10 and 11. Figure 1 shows the Schematic representation of the flow problem. The influence of magnetic factor $M$ on these velocity distributions is reflected in Figs. 2 and 3. It is witnessed in Fig. 2 that axial velocity distributions is declining with amassed estimations of magnetic factors. The intensification in $M$ indicates to expanding Lorentz force which is because of the interface of electric and magnetic fields in motion of electrically directed liquid. We can also say that bigger Lorentz force deals more resistance to the transportation phenomenon, that is why upsurge in $M$ agrees to a decline in velocity distributions. On the other hand a tangential component $g\left( \eta \right)$ is also declines with an expansion in estimations of $M$ as presented in Fig. 3. Figures 4 and 5 depict the influence of $\beta$ on $f^{\prime}\left( \eta \right)\,and\,g\left( \eta \right)$. We see from Figs. 4 and 5 that the $f^{\prime}\left( \eta \right)\,and\,g\left( \eta \right)$ decline against $\beta$. It is evident that for higher assessment of $\beta$ the viscosity of fluid improves and accordingly velocity $f^{\prime}\left( \eta \right)\,and\,g\left( \eta \right)$ increase. In fact, the second grade parameter $\beta$ improves the non-Newtonian behavior for its larger vales and consequently declines the velocity profile. The flow profiles in Fig. 6 suggest that augmentation in momentum limit layer viscosity declines the flow and gradient in flow of fluid. Moreover, the thicker the momentum limit layer proposes the little wall shear stress as a result of which $f^{\prime}\left( \eta \right)$ reduces with a corresponding increase in $\phi_{1} ,\phi_{2}$. Figure 7 shows the impact of $\phi_{2}$ on $g\left( \eta \right)$. The larger magnitude of the volume fraction $\phi_{1}$ and $\phi_{2}$ improve the resistive force and consequently declines the velocity field $g\left( \eta \right)$.

### Temperature profile

Figure 8 indicates that $\theta \left( \eta \right)$ reduces with $\Pr$ impact. It is additionally certain that expansion in the $\Pr$ prompts diminishing the limit of thermal layer thickness. Actually Prandtl number is inversely proportional to diffusivity of thermal boundary layer; hence increase in the values of $\Pr$ results in decline of thermal characteristics. So that $\theta \left( \eta \right)$ for extending sheet reduces by growing Pr. Figure 9 detects that the distribution $\theta \left( \eta \right)$ increases as $R$ upsurges with the datum that the rate of energy transmission jumps up due to increase in thermal radiations. Figure 10 observes that the temperature profiles $\theta \left( \eta \right)$ increase as $\theta_{w}$ increases because $\theta_{w}$ is the ratio of the hybrid nanofluid temperature at the sheet surface to the ambient temperature of the liquid. Figure 11 observes that the temperature profiles $\theta \left( \eta \right)$ increase as $\phi_{1} ,\phi_{2}$ increase. The larger magnitude of the parameters $\phi_{1} ,\,\,\phi_{2}$ enhancing the thermal efficiency of the base fluid and consequently the temperature profile increases.

### Table discussion

Table 2 shows the influence of various physical constraints on skin friction of the hybrid nanofluid flow. From Table 2 it is realized that the increase of the nanoparticle percentage in the base fluid boosts the skin friction of the base fluid. The parameters $S_{1}$ and $S_{2}$ are respiration parameters or injection and suction parameters of the nanofluid if we inject more fluid, then skin friction enhances. When we apply the orthogonal magnetic field to the flow of the hybrid nanofluid; this magnetic field attracts the metallic nanoparticles due to which the skin fraction increases. Skin friction enhances with the larger magnitude of the second grade parameter $\beta .$

**Table 2:** Outcome of different physical factors over Skin friction $C_{fx} Re_{x}^{\frac{1}{2}} = \frac{{\mu_{hnf} }}{{\mu_{nf} }}\left[ {f^{\prime\prime}\left( 0 \right) + \beta xg^{\prime\prime}\left( 0 \right)} \right]$.

$S1$	$S2$	$\beta$	$M$	$\phi_{1}$	$\phi_{2}$	$C_{fx} Re_{x}^{\frac{1}{2}}$
0.25	0.30	0.40	0.45	0.01	0.01	$0.4177710$
0.30						$0.4195709$
0.35						$0.4213785$
	0.30					$0.4177710$
	0.35					$0.4113878$
	0.40					$0.4049111$
		0.40				$0.4497204$
		0.45				$0.4497374$
		0.50				$0.4497888$
			0.40			$0.4565509$
			0.45			$0.4597204$
			0.50			$0.4628635$
				0.03		$0.4155745$
				0.04		$0.4166816$
				0.05		$0.4177710$
					0.03	$0.4193832$
					0.04	$0.42316718$
					0.05	$0.42839318$

Table 3 displays the influence of various physical factors on the Nusselt number. Nusselt number means the flow rate of the heat. From Table 3 we clearly see that if we enhance the Prandtl number then by physical definition of the Prandtl number when it enhances, the thermal conductivity of the fluid decreases, therefore the heat transfer rate declines with the enhancement of the Prandtl number. The addition in the thermal radiation factor improves the heat transfer rate. From Table 3 we clearly see that with the enhancement of the parameters $\phi_{1} ,\phi_{2} ,\theta_{w}$ improve the heat transfer rate. It has been observed that the hybrid nanofluids are the most efficient to improve the heat transfer rate as compared to the traditional fluids. Tables 4 and 5 are displayed to validate the obtained results with the existing literature. The Nusselt number and skin friction of the present study are compared and closed agreement is obtained.

**Table 3:** Influence of dissimilar physical constraints over Nusselt number $Nu_{x} Re_{x}^{{ - \frac{1}{2}}}$.

$\Pr$	$R$	$\theta_{w}$	$\phi_{1}$	$\phi_{2}$	$Nu_{x} Re_{x}^{{ - \frac{1}{2}}}$
10	0.3	0.2	0.05	0.03	$0.4877173$
11					$0.4827149$
12					$0.4773147$
	0.3				$0.4946563$
	0.6				$0.4957187$
	0.9				$0.4971463$
		0.2			
		0.4			
		0.6			
			0.05		
			0.06		
			0.07		
				0.03	
				0.04	
				0.05

**Table 4:** Comparison of the present work with the published work^15,16^.

	$C_{fx} Re_{x}^{\frac{1}{2}}$ [Results of Ref.^15^]	$C_{fx} Re_{x}^{\frac{1}{2}}$ [Results of Ref.^16^]	$C_{fx} Re_{x}^{\frac{1}{2}}$ [Present results]
0.4	0.4186600	0.4189483	
0.5	0.4437524	0.4438372	0.4428635
0.6	0.4726413	0.4727261	0.4717524
0.7	0.5015302	0.5015150	0.50028413

**Table 5:** Comparison of the present work with the published work^17^.

	$Nu_{x} Re_{x}^{{ - \frac{1}{2}}}$ [Results of Ref.^17^]	$Nu_{x} Re_{x}^{{ - \frac{1}{2}}}$ [Present results]
10	0.4889312	0.4877173
11	0.4867101	0.4827149
12	0.4745012	0.4773147

## Conclusions

The magnetohydrodynamic second-grade hybrid nanofluid flow towards an extending/shrinking sheet with thermal radiation is inspected in this investigation. The main concern of this research work is to consider the hybrid nanofluid which is perceived by hanging two distinctive nanoparticles known as alumina and copper within the second grade fluid while the fluid motion is formed from the non-linearly stretching/shrinking sheet.

The important observations are given below.

- Second-grade fluid used as a base fluid for the solid nanoparticles and the influence of the second grade parameter *β* observed versus the velocity field.
- $Al_{2} O_{3}$ and $Cu$ are used as the solid nanoparticles. The increments in the volume fraction $\phi_{1}$ and $\phi_{2}$ of the nanoparticles increase the thermal efficiency of the fluid.
- For greater values of *M* the velocity ${f}' (\eta )$ and ${g} \, (\eta )$ decrease.
- The heat transfer rate upsurges for bigger *Rd* and $\theta_{w} \,$.
- The heat transfer rate diminishes with augmentation of Prandtl number.
- The velocity profile declines for the larger magnitude of the Reynolds number.
- It has been observed that the hybrid nanofluids are most efficient to enhance the thermal conductivity of the second grade fluids as compared to the traditional fluids.

## Data Availability

The data that support the findings of this study are available from the corresponding author upon reasonable request.

## Abbreviations

$$u\,,\,v$$: Velocity components
$$x\,,\,y$$: Cartesian coordinates
$T$: Hybrid nanofluid temperature
$$\lambda$$: Constant parameter
$$T_{\infty }$$: Ambient temperature
$$M$$: Magnetic parameter
$$T_{w}$$: Wall temperature
$$\beta_{1}$$: Dimensionless parameter
$\theta_{w} \,$: Temperature ratio parameter
$\rho_{hnf}$: Density of hybridnanofluid
$\nu_{hnf}$: Kinematic viscosity of hybridnanofluid
$\alpha_{hnf}$: Thermal diffusivity of hybridnanofluid
$\left( {\rho c_{p} } \right)_{hnf}$: Heat capacity of hybridnanofluid
$$S_{1} ,S_{2}$$: Transpiration parameters
$$Ec$$: Eckert number
$$R$$: Radiation parameter
$C_{fx}$: Skin friction coefficient
$$\tau_{w}$$: Shear stress
$B_{0} \,$: Strength of magnetic field
$\beta$: Second grade fluid parameter
$$Nu_{x}$$: Nusselt number
$$Re_{x}$$: Reynolds number
$$\Pr$$: Prandtl number
$L$: Characteristic length of the sheet
$$f^{\prime}$$: Dimensionless velocity
$$\infty$$: Ambient condition
$w$: Condition on surface
$^{\prime}$: Derivative with respect to $\eta$
